# Lateral Subcutaneous Locking Compression Plate and Small Incision Reduction for Distal‐third Diaphyseal Humerus Fractures

**DOI:** 10.1111/os.12398

**Published:** 2018-08-28

**Authors:** Hong Chang, Zi‐Long Yao, Yi‐Long Hou, Yang Cao, Xin‐Hui Guo, Guan‐Jun Li, Bin Yu

**Affiliations:** ^1^ Department of Orthopaedics and Traumatology Nanfang Hospital, Southern Medical University Guangzhou China; ^2^ Department of Orthopaedics 421 Hospital of PLA Guangzhou China

**Keywords:** Humeral fracture, Locking compression plate, Small incision reduction, Subcutaneous

## Abstract

**Objective:**

Iatrogenic radial nerve injury is a great challenge for orthopaedic surgeons who deal with distal‐third diaphyseal humerus fractures. Conventional open reduction and internal fixation (ORIF) remains the gold standard, but complications such as nonunion and iatrogenic radial nerve injury still occur. We fixed the fractures with a lateral locking compression plate (LCP) subcutaneously after small incision reduction to protect the radial nerve. This study reports the clinical and radiographic outcomes of our modified method.

**Methods:**

Thirty‐eight patients with distal‐third diaphyseal humerus fractures were treated with lateral subcutaneous LCP and small incision reduction at our department between September 2013 and August 2016. There were 33 males and 5 females, with an average age of 30.3 years (range, 17 to 49 years). All the cases were types A or B (AO/OTA classification, type A, 24 cases; type B, 14 cases). Among them, 6 cases were combined with preoperative radial nerve palsy. All patients were diagnosed with closed humeral fractures after X‐ray examination, and had typical upper limb pain, swelling, and movement disorders. The operations were performed by a single surgeons’ team. Union time, range of motion (ROM), University of California, Los Angeles (UCLA) shoulder rating scale, and Mayo Elbow Performance Index (MEPI) scores were assessed to evaluate the postoperative results.

**Results:**

All patients were followed up for an average of 11.4 months (range, 3 to 36 months). The average operation time was 75.5 min (range, 60 to 150 min) and average intraoperative radiation exposure was 10.5 s (range, 8 to 18 s). Bony union was achieved in all cases after an average of 16.2 weeks (range, 12 to 25 weeks). No complications such as infection or screw and plate fracture occurred, and no iatrogenic radial nerve injury was observed. According to the UCLA shoulder rating scale, the average score was 33.7 (range, 31 to 35), with 33 excellent (86.8%) and 5 good cases (13.2%). They were all excellent according to their MEPI scores (ranging, 94 to 100, with an average of 97.4). The average operation time for secondary removal of the plate was 15.2 min (range, 10 to 20 min), and no complications such as infection or secondary radial nerve injury occurred.

**Conclusions:**

Lateral subcutaneous LCP and small incision reduction may reduce the risk of iatrogenic radial nerve injury significantly in the treatment of distal‐third diaphyseal humerus fractures. It also leads to solid fixation, good postoperative function, and convenient removal of the plate without injuring the radial nerve.

## Introduction

Distal‐third diaphyseal humerus fractures are commonly treated by open reduction and internal fixation (ORIF), intramedullary nail fixation (IMN), external fixation and minimally invasive plate osteosynthesis (MIPO)^1^, ^2^, ^3^, ^4^. ORIF is usually regarded as the gold standard treatment and, thus, is the most commonly‐used approach in the clinic because of the anatomical reduction and less interference with the elbow and shoulder function^5^, ^6^, ^7^, ^8^, ^9^. In the patients accepting ORIF, the anterolateral approach is used most commonly, but its extensive stripping of soft tissue and periosteum might impair blood supply to the fracture sites and influence the fracture healing^10^. Relevant published studies report that the rate of nonunion after ORIF could reach up to 5%–10%^8^, ^11^, ^12^.

Moreover, the incidence of radial nerve paralysis is the highest (up to 29%) in humeral fractures^13^ and the risk of iatrogenic radial nerve injury may be increased in the treatment with plate fixation because the radial nerve is close to the bone surface of the distal‐third diaphyseal humerus^1^, ^14^. Furthermore, the more important and unfavorable factor is the exposure of the radial nerve in the operation. Even though surgeons are very careful to dissect and protect the nerve, the risk of iatrogenic injury remains fairly high (up to 17.6%)^13^, ^15^. If a secondary operation is needed to manage such complications as non‐union or to remove an internal fixation device, exposure of the surgical area may be very difficult because of the adhesion of scarred tissue and, thus, greatly increased risk of iatrogenic radial nerve injury^16^. Some surgeons choose nonoperative treatment with a functional brace or retain the plate in the patient’s body permanently after ORIF to decrease the risk of iatrogenic radial nerve injury. Compared with middle and proximal‐shaft humerus fractures, distal‐third diaphyseal humerus fractures may impose a greater challenge on orthopaedic surgeons^17^.

The IMN technology required sufficient bone mass of the distal of the fractures and enough effective area for fixation, as well as appropriate surgical experience. Moreover, the complication ratio of IMN is much higher than that of plate fixation, but there was no significant difference in shoulder and elbow function scores and bone healing rates postoperatively. Hence, the use of IMN for treatment of distal‐third diaphyseal humerus fractures in the clinic is not extensive^18^, ^19^.

External fixation technology is also commonly used by orthopaedic surgeons to treat humeral fractures, but it is more often used as a temporary fastening device or in case of emergency. Furthermore, the external fixation can be associated with problems such as aseptic loosening and infection of the screw channel, so it is used less as the terminal treatment for closed humeral fractures^4^, ^20^, ^21^.

Anterior MIPO technology for the treatment of humeral fractures has been used extensively in recent ten years. Some scholars consider it to have many advantages, such as being minimally invasive, as well as being associated with less bleeding, shorter operative time, and lower risk of radial nerve injury, and regard it as a potential substitute method for conventional ORIF^22^, ^23^, ^24^, ^25^, ^26^. Although the anterior MIPO technology has many advantages, some negative factors should still be taken into consideration. Postoperative malreduction and malrotation of MIPO can result in internal fixation failure or joint function restriction, and is correlated with subsequent long‐term shoulder degeneration^27^. For the treatment of distal‐third diaphyseal humerus fractures, MIPO technology encounters the problem of inadequate fixation space^28^. Moreover, in using the anterior approach of MIPO, the injury of the muscle tissue would affect the elbow and lead to the delay of functional recovery^29^. Although using the anterior approach of MIPO avoids the radial nerve, the distance between the nerve and plate remains very small (average, 4 mm), especially in the transition between the third and fourth quarters of the humeral shaft^30^. Therefore, the anterior MIPO technology may still not achieve the aim of the nerve being unaffected completely.

In the last 10 years, with the wide application of locking compression plate (LCP) technology, some scholars have attempted to use LCP as an external fixator to treat extremity fractures. It seems an attractive technique to deal with fractures involving complex wounds or to improve the tolerability of treatment. It has been reported that externalized LCP could act an alternative to the unilateral external fixator (UEF) for treating distal tibial fractures, and the axial and torsional stiffness are not compromised^31^, ^32^. Although this new technique still has some deficiencies, such as potential screw channel infections and inconvenience for nurses, it provides us with a new concept for clinical treatment of distal‐third diaphyseal humerus fractures.

To obtain effective stabilization, good functional recovery, convenient removal of the plate, and superior protection of the radial nerve, we devised a new surgical method for distal‐third diaphyseal humerus fractures. We used LCP to fix the fracture through a lateral approach subcutaneously after small incision reduction in a series of 38 patients from September 2013 to August 2016.

This study evaluates the clinical and radiological outcomes of this series of patients with distal‐third diaphyseal humerus fractures, and summarizes and analyzes the associated factors that influence the surgery. We also examined, for instance, the incidence of radial nerve injury and other related complications during the perioperative period, the amount of intraoperative blood loss and the time taken for secondary removal of the internal plant. At the same time, we evaluated the postoperative functional recovery of shoulder and elbow joints to understand the therapeutic effect of this modified method and to provide a clinical basis for further promotion and application of the treatment approach.

## Patients and Methods

### *Clinical Data*

**Figure 1:**
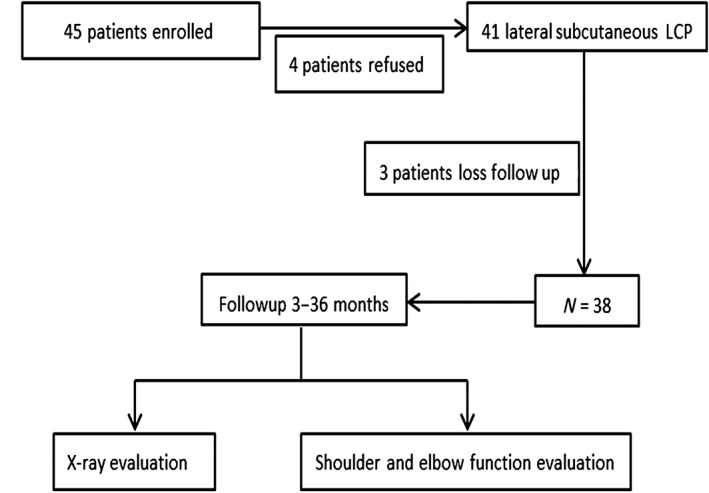
Flowchart of the selection process and follow‐up for the study. LCP, locking compression plate.

This study was a retrospective review of the medical records of 45 patients with distal‐third diaphyseal humerus fractures who had been treated at the author’s (C. Hong’s) institution between September 2013 and August 2016. Of these patients, 4 were treated with conventional ORIF and 41 with lateral subcutaneous LCP and small incision reduction; 38 of these had complete follow‐up data (Fig. 1). They were 33 males and 5 females, aged from 17 to 49 years (mean ± SD, 30.3 ± 9.4 years). The average body mass index (kg/m^2^) of the patients was 21.5 (range from 16 to 27), and 19 patients (50%) were habitual smokers. The minimum duration of follow‐up was 3 months (mean ± SD, 11.4 ± 5.3 months; range from 3 to 36 months). Six patients had preoperative radial nerve palsy.

All operations were performed by the same team of surgeons with adequate clinical experience in orthopaedic traumatology. Inclusion criteria were as follows: (i) simple distal‐third diaphyseal humerus fractures defined according to AO/OTA classification^33^, with all the cases being of types A and B (type A, 24 cases: A1, 16 cases, A2, 7 cases, A3, 1 case; type B: 14 cases, B1, 7 cases, B2 4 cases, B3 3 cases), including 27 cases of spiral fracture caused by throwing activity (71.1%), 5 cases caused by falling down (13.1%), 5 cases caused by other damage (13.1%), and only 1 transverse fracture (2.6%), which was caused by a traffic accident; (ii) distal‐third diaphyseal humerus fractures at least 5‐cm proximal to the olecranon fossa; (iii) age older than 15 years with completion of bone growth; and (iv) acceptance of the surgical program preoperation. Exclusion criteria were as follows: (i) open fractures, pathologic fractures and comminuted fractures (AO/OTA classification types C); (ii) the fracture line extends to the olecranon fossa or at a distance from olecranon fossa less than 5 cm; (iii) children and teenagers under 15 years of age; and (iv) patients who had refused to accept the surgical program. This study was approved by the institutional review board and informed consent was obtained from the patients.

### *Preoperative Treatment*

All patients were hospitalized to receive surgical treatment. Preoperatively, the fractured upper limb was immobilized with plaster to stabilize the fracture site and avoid secondary radial nerve paralysis. The injured upper limb was lifted on a pillow, with ice compression applied to reduce swelling. The plaster must be checked periodically to avoid complications such as osteofascial compartment syndrome. All the patients were not allowed to eat and drink 8 h before surgery. The skin of the injured upper arm was cleaned with normal saline and alcoholic solution beforehand. Intravenous antibiotics were used 0.5 h before surgery to prevent infection.

### *Surgical Techniques*

The operation was carried out under continuous interscalene brachial plexus block or general anesthesia. The patient was placed in a supine position and the fractured arm on a radiolucent board, keeping the limb in abduction and the elbow flexed at 60°‐90°. The small incision surgical approach for fracture reduction is described by Lee *et al*.^28^. Briefly, an anterolateral incision of 3–5 cm was made on the surface of the lateral upper arm corresponding to the fracture location. Dissection was then performed at the lateral aspect of biceps brachialis down to the brachialis. The longitudinal separation at the outer third of the brachialis was executed. The humerus was exposed after pull the biceps brachialis and the inside of the two‐thirds of the brachialis.

Care was taken to avoid damaging the forearm lateral cutaneous nerve and muscle cutaneous nerve in the process of the separation. The radial nerve was then touched between the brachialis and brachioradialis. In most cases, neurolysis for the radial nerve was not necessary unless palsy of the radial nerve existed preoperatively. The radial nerve was protected when the outer third of the brachialis and brachioradialis was pulled to expose the fracture. Basic fracture principles were followed, and reduction was achieved by opening the fracture site with a minimum of soft tissue stripping. In this process, the operator should reduce stripping of the periosteum and brachialis muscle as far as possible to decrease adhesion of scarred tissue and to help recovery after surgery. Kirschner wires (ϕ 2.0 mm) were used to fix the fracture temporarily, which facilitated the next steps of the operation. Afterwards, two incisions of 3 cm at the proximal and distal ends were made on the surface of the lateral upper arm, respectively, after good reduction of the fracture was confirmed by the intraoperative real‐time fluoroscopy, using a periosteal elevator to make a tunnel to connect the two incisions through the blunt stripped subcutaneous tissue from the proximal to the distal window. Then a 4.5‐mm LCP (Vortex plate system, Sanatmetal, Hungary) was inserted from the distal to the proximal window through the subcutaneous tunnel. Considering that the increased perpendicular distance between the plate and the bone surface would affect the mechanical stability, we used suitable plates that were relatively long and inserted at least three 4.5‐mm locking screws between both ends of the fracture.

It should be confirmed that the plate position matches the bone before the insertion of screws. We drilled two Kirschner wires (ϕ 2.0 mm) into the cortex through the second proximal hole and the second distal hole, respectively, for temporary positioning. After a satisfactory plate position was confirmed by fluoroscopy, according to the standard techniques, two 4.5‐mm locking screws at the proximal and distal ends of the plate were inserted, respectively. The operator must be careful when inserting the most distal screw to avoid penetration of the screw into the olecranon fossa. A short unicortical screw was used when a potential risk might exist. After preliminary stability was achieved, the two Kirschner wires used for temporary positioning were removed. The last two screws were then implanted through the two locking holes which remained after the Kirschner wires were removed.

**Figure 2:**
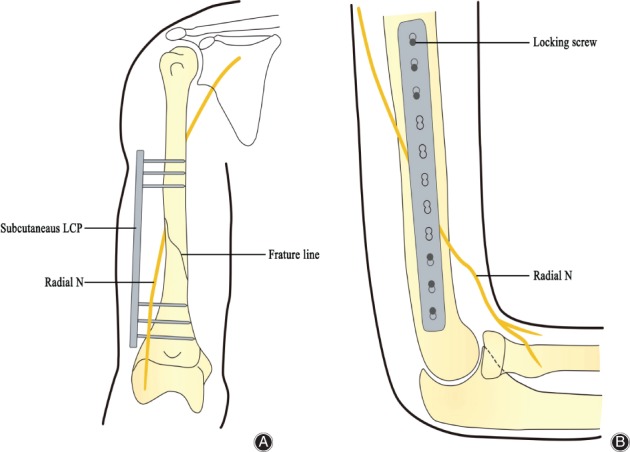
The abridged general view of the distal‐third diaphyseal humerus fracture fixed with lateral subcutaneous locking compression plate (LCP). (A) The anteroposterior view shows the relationship between the plate, the fracture line, and the radial nerve; the LCP is located subcutaneously and is not in contact with the radial nerve. (B) The radial nerve near the bone surface at the distal‐third diaphyseal humerus and below the plate; the locking screws are kept away from the radial nerve.

**Figure 3:**
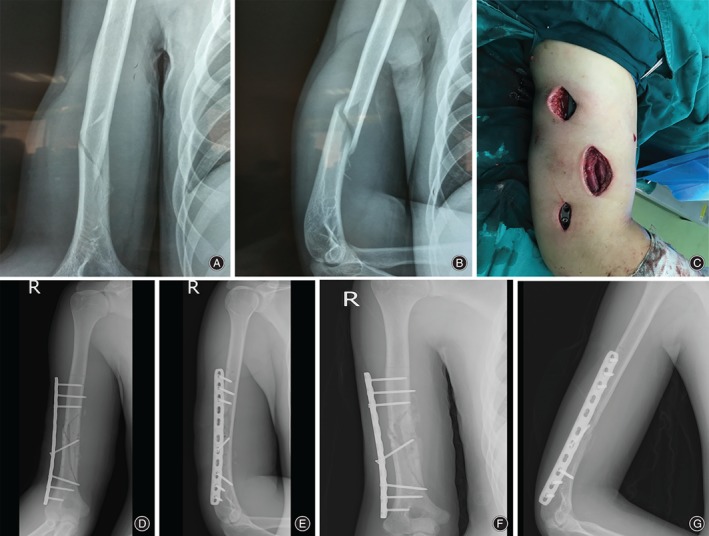
Preoperative anteroposterior (A) and lateral (B) X‐ray films of a distal‐third diaphyseal humerus fracture. The operative incisions and LCP inserted through the subcutaneous tunnel (C). Anteroposterior (D) and lateral (E) X‐ray films 3 days after surgery show that the fracture achieved good reduction. Anteroposterior (F) and lateral (G) X‐ray films at the 6‐week follow‐up showing well‐maintained linear and positional alignments of the fracture and obvious callus formation at local regions without any implant failure.

Throughout the process the screws should be kept away from the radial nerve. LCP was placed above the lateral muscles at a suitable distance from the bone to avoid affecting muscular activity. The distance between the bone surface and the plate was determined by the muscle thickness of the patient (Fig. 2). For fractures of types A and B combined with a long oblique and spiral fracture, a 3.5‐mm cortical screw was used to assist plate fixation. Before the end of the operation, fracture reduction and fixation stability must be confirmed by intraoperative fluoroscopy (Fig. 3).

**Figure 4:**
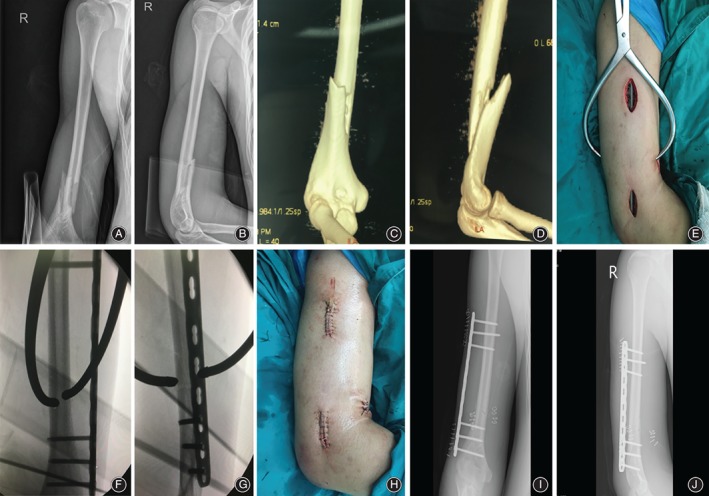
Preoperative anteroposterior (A) and lateral (B) X‐ray films of a distal‐third diaphyseal humerus fracture. The preoperative CT three‐dimensional reconstruction (C, D) shows obvious fracture displacement. During the surgery, the forceps were used to assist the fracture reduction through two small percutaneous incisions at anterior and posterior, and LCP was inserted lateral subcutaneously through two small incisions (E). Anteroposterior (F) and lateral (G) intraoperative fluoroscopy after fixation shows that the fracture achieved good reduction and the LCP was unattached to the bone surface. The incisions after suture were small (H). Anteroposterior (I) and lateral (J) X‐ray films 3 days after surgery shows that the fracture was in good position and firmly fixed.

For some type A fractures, we used forceps to assist fracture reduction through two percutaneous small incisions at the anterior and posterior aspects of the distal upper arm, respectively. Care was taken to avoid injury to the blood vessels located at the anteromedial aspect. The reduction forceps were used to ensure stability of the fracture site. After the linear and positional alignments were confirmed by fluoroscopy, the techniques of lateral subcutaneous LCP were used to fix the fracture as mentioned above (Fig. 4).

### *Postoperative Care*

Prophylactic intravenous antibiotics were routinely given for 24 h postoperatively. The incisions were kept clean and dry until they healed completely. The incision stitches were taken out 10–12 days after surgery. The elbow was trained to flex and extend passively three times every day from the third day after operation. Active muscle contraction training for the forearm and hand should be started as early as possible after surgery to promote swelling subsidence. The magnitude of functional training of the elbow gradually increased and the rehabilitative training of the shoulder also began progressively 2 weeks after surgery. At 6 weeks, active strength training was intensified until the fracture was healed.

### *Function Evaluations*

The injured upper arms of the patients were immobilized in a sling for 4 weeks postoperatively. All patients were re‐examined with X‐rays (anterior–posterior and lateral radiographs) to evaluate the functional recovery of the shoulder and elbow at out‐patient departments at postoperative 6 weeks, 12 weeks and 6 months, respectively. The range of motion of the joints (shoulder and elbow) was assessed by one of the authors (GJ Li) at each follow‐up visit using a standard goniometer. The degree of intorsion rotation was measured with the upper arm in 90° abduction. The function of the shoulder was evaluated using the University of California, Los Angeles (UCLA) shoulder rating scale^34^ (range from 0 to 35); the elbow function was evaluated using the Mayo Elbow Performance Index (MEPI) score^35^ (range from 0 to 100). Fracture union was defined as presence of bridging callus radiographically visible on at least three cortices; non‐union was defined as absence of healing 6 months after surgery.

## Results

### *General Results*

For the 38 patients treated with lateral subcutaneous LCP fixation, the interval from injury to operation averaged 3.6 days (range from 0.5 to 7 days), operation time 75.5 min (range from 60 to 150 min) and time for intraoperative radiation exposure 10.5 s (range from 8 to 18 s). All the patients obtained bony union uneventfully. The average union time was 16.2 weeks (range from 12 to 25 weeks). The internal fixation devices were removed in all 38 patients when the unions of fractures were observed. The average operation time for secondary removal of the plate was 15.2 min (range from 10 to 20 min).

### *Clinical Outcomes*

Twelve weeks after surgery, the average range of motion (ROM) of the shoulder was: 174.5° of rising (range from 165° to 180°), 63.5° of extorsion rotation (range from 45° to 80°), and 72° of intorsion rotation (range from 50° to 80°). According to the UCLA shoulder rating scale, the average score was 33.7 (range from 31 to 35), giving 33 excellent cases (86.8%) and 5 good cases (13.2%).

The average ROM of the elbow: 131.5° of flexion (range from 120° to 145°) and 3° of extension (range from 0° to 15°). The average MEPI score was 97.4 (range from 94 to 100) and all were rated as excellent.

### *Complications*

Local dissolution of the subcutaneous fat occurred postoperatively in 3 patients whose incision healed by second intention after dressing change. There were no cases of infection. In 6 patients who had suffered preoperative radial nerve palsy, the radial nerve was dissected and separated for neurolysis during surgery. Through intraoperative exploration we found that no nerve was broken or tore; the nerve pull and compression caused by the fracture end are common factors that might lead to paralysis of the radial nerve. To protect the radial nerve and avoid secondary damage, some methods can be used such as temporary fixation of the fracture, gentle implantation of the plate during surgery, and placement of the screws as far as possible away from the anatomical position of the radial nerve. Using reduction of fracture and neurolysis, the nerve function was recovered 4 months after the operation. All patients had good alignment, and no malunion, nonunion or delayed union was observed. No complications such as screw and plate fracture occurred. No patient suffered postoperative radial nerve injury or secondary radial nerve injury in the process of removal of the internal fixation devices.

## Discussion

It is reported that in distal‐third diaphyseal humerus fractures of type A or B (AO‐OTA) the nonunion rate seems to be higher^36^, ^37^ and operative treatment would achieve more predictable alignment and potentially quicker return of function. Therefore, surgical treatment is recommended. Because ORIF with a dynamic compression plate (DCP) as the current standard has the advantages of direct reduction and absolute stability, it is the more common surgical option^1^. Iatrogenic radial nerve injury, however, one of the major complications of this surgical method, perplexes the orthopedists profoundly. Exploration of the radial nerve is needed, whether through the lateral or posterior approach, and the internal device is placed directly under the nerve. This may lead to adhesion of scarred tissue and fibrosis around the plate and the radial nerve after surgery, increasing the risk of secondary radial nerve injury^15^, ^28^, ^38^.

### *Advantages of Modified Method*

To solve the above problems, we designed a surgical method that can obtain effective reduction and fixation of the fracture, avoid aggravation of the radial nerve, reduce stripping of the muscles and periosteum, and allow convenient removal of the internal fixation device after bone union.

In the technique “supercutaneous plating” by Kloen^39^, the LCP can be used as an external fixator for treatment of distal tibial fractures and humeral infected nonunion^40^, ^41^. In a biomechanical comparative study of axial and torsional stiffness by Ang *et al*.^31^, LCP was used as an external fixator in comparison with UEF. No statistical evidence was found that LCP was better than UEF regarding the axial stiffness and the torsional stiffness.

Therefore, we supposed that LCP might be used subcutaneously to treat humeral fractures. Consequently, we designed a surgical method that uses a small anterolateral incision to reduce the fracture before an LCP and locking screws are implanted to fix the fracture via two small lateral incisions that connect a subcutaneous tunnel. This method might realize fixation *in vivo* using LCP as the principle of external fixation and avoiding drawbacks of an external fixator such as infection of screw channels and inconvenience for nurses. Meanwhile, aggravation of the radial nerve can be avoided completely because the plate and the nerve do not touch any more.

### *Operative Time and Complication Rates*

Our method, lateral subcutaneous LCP and small incision reduction, can decrease surgical injury and is relatively safe. In the operation, the outer third of the brachialis muscle and brachioradialis muscle are pulled together to protect the radial nerve, which needs not be dissected unless the fracture is complicated with preoperative radial nerve palsy. This will significantly shorten the operation time if the operator is familiar with the anatomic structures. Kim *et al*. reported that two groups of patients with humeral fracture (AO/OTA, types A and B) were treated with ORIF and MIPO techniques, respectively; the operation time was approximately 105–116 min, and there was no significant difference between the two groups^42^. However, in this group of patients who used the modified method, the average operative time was 75.5 min, which was lower than that reported.

Furthermore, the lateral subcutaneous LCP technique could reduce the complication rate of radial nerve injury compared with traditional ORIF or IMN. Lim *et al*. reported 170 cases of humerus fractures treated with ORIF; the incidences of postoperative wrist drop were high at 17.6%, and the lower third fractures had the highest incidence of iatrogenic radial nerve injury^13^. However, for the modified method, because there are no important nerves or vessels at the lateral aspect of the upper arm and it is not necessary to place the plate close to the bone and the screws are kept away from the anatomical position of the radial nerve, the operation is relatively safe. Therefore, our method helps decrease the aggravation to the radial nerve and, thus, reduces the risk of nerve injury. In this series of 38 patients, no radial nerve injury was observed postoperatively except in the 6 cases who had suffered preoperative radial nerve palsy. Quite a number of patients with humeral fractures require removal of the internal fixation device. Scarred tissue adhesion around the plate and the radial nerve, however, makes the surgery more difficult and greatly increases the operation time, and the risk of secondary injury to the radial nerve rises obviously^16^. However, the subcutaneous LCP technique might solve these problems satisfactorily. Because all the surgical procedures are done in the subcutaneous tunnel, deep tissue does not need to be taken into account. In the secondary surgery to remove the internal fixation devices after fracture healing, convenient operation steps may not aggravate the radial nerve and muscles, reducing operation time and avoiding the risk of radial nerve injury completely. In our patients, the time for secondary removal averaged only 15.2 min and no secondary radial nerve injury occurred.

### *Postoperative Recovery and Function*

In addition, the two anterolateral incisions for reduction of the fracture are minimally invasive because they are small and only a little periosteum is detached. This protects the blood supply around the broken ends of the fracture maximally, promoting fracture healing. As a result, all the fractures in this study healed successfully, without any internal fixation failure. Furthermore, our subcutaneous LCP not only uses the principle of an external fixator to realize the goal of fixation *in vivo* but also has the benefit of promoting fracture healing through dynamic fixation^41^.

Our method may also lead to satisfactory functional recovery of the shoulder and elbow. According to related reports, Patino summarized the clinical data of 30 patients with humeral fractures treated with antegrade IMN and concluded that decreased shoulder ROM was common postoperation^43^. Moreover, Kobayashi *et al*. reported a series of patients with humeral fractures treated with MIPO; the median time to normal motion recovery was 19 days in the shoulder, and 60 days in the elbow^29^. They considered that elbow function requires longer recovery time than the shoulder, and this may be due to the distal approach. However, the modified method almost avoids the effects on the shoulder and elbow joints completely, and the small incision reduction technique also could reduce muscle and soft tissue injuries, and it is helpful for rapid postoperative recovery. In this study the UCLA and MEPI scores of the patients treated with the lateral subcutaneous LCP technique were excellent or good, and ROM of the shoulder and elbow joints were almost normal 6 weeks after surgery. This could be because the damage to the muscles by screws is very limited and all the screws are implanted through locking sleeves. Moreover, because the LCP is completely in the body, our method may also improve quality of life and satisfaction of the patients, and facilitate care of incisions as well.

### *Limitations*

Nevertheless, our method has drawbacks that need to be improved. Although fracture reduction can be obtained via a small incision, the periosteum and muscles cannot be fully protected. Besides, it remains unclear whether the subcutaneous LCP can provide sufficient stiffness to maintain fracture fixation and, thus, further biomechanical research is needed. For the distal‐third diaphyseal humerus fractures that are not complicated with preoperative radial nerve palsy, closed or percutaneous reduction may be preferable to reduce possible iatrogenic damage and operation time. It is also helpful to use an auxiliary device in the process of surgery, such as traction through olecranon or an external fixator to assist traction^44^.

### *Conclusion*

Our lateral subcutaneous LCP and small incision reduction is a safe and efficient procedure for distal‐third diaphyseal humerus fractures. Adequate stabilization and satisfactory functional recovery of the upper arm can be obtained. The risk of iatrogenic radial nerve injury is low if surgical techniques are appropriate. Furthermore, internal fixation devices can be easily taken out from the body without aggravating the radial nerve, greatly reducing the risk of secondary injury to the radial nerve. The optional surgical removal of implants may meet the requirement of most patients after the fracture is healed.
